# Cardiovascular risk quantification using QRISK-3 score in people with intellectual disability

**DOI:** 10.1192/bjo.2021.187

**Published:** 2021-06-18

**Authors:** Jamie Sin Ying Ho, Vikram Rohra, Laura Korb, Bhathika Perera

**Affiliations:** 1 Haringey Learning Disabiliy Partnership, Barnet, Enfield an Haringey Mental Health Trust; 2 North Middlesex University Hospital NHS Trust

## Abstract

**Aims:**

The prevalence of cardiovascular diseases (CVD) in people with intellectual disability (ID) is around 14%, higher than the general population. However, CVD risk assessments are not consistently performed. Given the high risk of premature deaths in people with ID, it is important to identify preventable risk factors and follow evidence-based interventions. QRISK-3 is a validated risk-stratification tool, which calculates the 10-year risk of developing a heart attack or stroke (https://qrisk.org/three/index.php). There are no published studies on the use of QRISK-3 in people with ID. This project aimed to understand the use of QRISK-3 in an ID clinic and to quantify individual CVD risks to recommend appropriate management options.

**Method:**

A cross sectional study was performed on 143 patients open to an ID psychiatry clinic. Patients and carers were sent an accessible information leaflet on this study. Basic demographic data and information on psychiatric diagnoses were collected. Patients were grouped according to the presence of severe mental illness (SMI) defined as schizophrenia, bipolar disorder and other psychotic illnesses. QRISK-3 ≥ 10% was defined as elevated risk in accordance with NICE guidelines. Patients who had a high QRISK-3 score were advised to contact their GP.

**Result:**

Of 143 patients, 73 (51.0%) had a mild ID and the remaining had a moderate to severe ID. The mean age was 43.3 years, 53.1% were male. Overall, 28 (19.6%) participants had an elevated CVD risk, of whom 16 (57.1%) were not on statins, which is the recommended treatment. The mean QRISK-3 score was 6.31 (standard deviation [SD] 8.95), and the relative risk is 3.50 (SD 7.13). The proportion of QRISK-3 ≥ 10% and mean score were not significantly different in those with SMI, but those with SMI were more likely to be prescribed statins than those without (14 [31.1%] vs 10 [10.2%], p = 0.002). Statins were given to 24 (16.8%) participants, of whom 12 (50%) had elevated CVD risk. 89% had a blood pressure recording within the past 5 years, 87% had height and 88% had weight recorded. 73% had lipid serology results recorded.

**Conclusion:**

Elevated CVD risk was common in this ID study population, and more than half with elevated QRISK-3 were not on the medical treatment recommended by national guidelines. QRISK-3 could feasibly be implemented in the outpatient setting. Increased routine CVD risk assessment and management should be considered as another measure to reduce morbidity and mortality.

